# Risk of De Novo Hypertensive Disorders of Pregnancy After Exposure to PM1 and PM2.5 During the Period From Preconception to Delivery: Birth Cohort Study

**DOI:** 10.2196/41442

**Published:** 2023-01-23

**Authors:** Zhichao Yuan, Hai-Jun Wang, Qin Li, Tao Su, Jie Yang, Junjun Chen, Yuanzhou Peng, Shuang Zhou, Heling Bao, Shusheng Luo, Hui Wang, Jue Liu, Na Han, Yuming Guo, Yuelong Ji

**Affiliations:** 1 Department of Maternal and Child Health School of Public Health Peking University Beijing China; 2 National Health Commission Key Laboratory of Reproductive Health Beijing China; 3 Tongzhou Maternal and Child Health Care Hospital of Beijing Beijing China; 4 Department of Electrical and Computer Engineering Whiting School of Engineering Johns Hopkins University Baltimore, MD United States; 5 Department of Epidemiology and Biostatistics School of Public Health Peking University Beijing China; 6 Department of Epidemiology and Preventive Medicine School of Public Health and Preventive Medicine Monash University Melbourne Australia

**Keywords:** air pollution, PM_2.5_, PM_1_, hypertensive disorders of pregnancy, preconceptional period, lag effect, pregnancy, hypertension, hypertensive disorders, risk, pollutants, exposure, maternal health, perinatal health, pollution

## Abstract

**Background:**

Particulate matter (PM) is detrimental to the respiratory and circulatory systems. However, no study has evaluated the lag effects of weekly exposure to fine PM during the period from preconception to delivery on the risk of hypertensive disorders of pregnancy (HDPs).

**Objective:**

We set out to investigate the lag effect windows of PM on the risk of HDPs on a weekly scale.

**Methods:**

Data from women with de novo HDPs and normotensive pregnant women who were part of the Peking University Retrospective Birth Cohort, based on the hospital information system of Tongzhou district, were obtained for this study. Meteorological data and data on exposure to fine PM were predicted by satellite remote sensing data based on maternal residential address. The de novo HDP group consisted of pregnant women who were diagnosed with gestational hypertension or preeclampsia. Fine PM was defined as PM_2.5_ and PM_1_. The gestational stage of participants was from preconception (starting 12 weeks before gestation) to delivery (before the 42nd gestational week). A distributed-lag nonlinear model (DLNM) was nested in a Cox regression model to evaluate the lag effects of weekly PM exposure on de novo HDP hazard by controlling the nonlinear relationship of exposure–reaction. Stratified analyses by employment status (employed or unemployed), education level (higher or lower), and parity (primiparity or multiparity) were performed.

**Results:**

A total of 22,570 pregnant women (mean age 29.1 years) for whom data were available between 2013 and 2017 were included in this study. The prevalence of de novo HDPs was 6.7% (1520/22,570). Our findings showed that PM_1_ and PM_2.5_ were significantly associated with an elevated hazard of HDPs. Exposure to PM_1_ during the 5th week before gestation to the 6th gestational week increased the hazard of HDPs. A significant lag effect of PM_2.5_ was observed from the 1st week before gestation to the 6th gestational week. The strongest lag effects of PM_1_ and PM_2.5_ on de novo HDPs were observed at week 2 and week 6 (hazard ratio [HR] 1.024, 95% CI 1.007-1.042; HR 1.007, 95% CI 1.000-1.015, respectively, per 10 μg/m^3^ increase). The stratified analyses indicated that pregnant women who were employed, had low education, and were primiparous were more vulnerable to PM exposure for de novo HDPs.

**Conclusions:**

Exposure to PM_1_ and PM_2.5_ was associated with the risk of de novo HDPs. There were significant lag windows between the preconception period and the first trimester. Women who were employed, had low education, and were primiparous were more vulnerable to the effects of PM exposure; more attention should be paid to these groups for early prevention of de novo HDPs.

## Introduction

A risk of multiple adverse health outcomes is attributable to increased exposure to the ambient particulate matter (PM). Fine PM is defined as ambient particles with an aerodynamic diameter ≤2.5 μm or ≤1 μm, that is, PM_2.5_ and PM_1_, respectively. PM is derived from a wide range of sources, such as forest fires, coal combustion, industrial processes, and traffic emissions [1]. It is composed of various toxic pollutants, including organic carbon, alkanes, metals, sulfates, and nitrates [2,3]. When people inhale the air, fine PM can easily translocate the abovementioned toxic pollutants across biological membranes from the pulmonary alveoli to the blood circulation, further resulting in systemic and tissue injury due to its physical characteristics of tiny size and large surface area [4]. According to the World Health Organization (WHO) air quality guidelines, about 92% of people in the world are exposed to excessive fine PM. Not long ago, the WHO updated the limit on exposure to fine PM, decreasing it from 10 μg/m^3^ to 5 μg/m^3^, based on research findings from the past 15 years. PM exposure is regarded as a risk factor for cardiovascular diseases and adverse perinatal outcomes [5,6]. An accumulating number of studies have used in vitro and in vivo experiments to show that fine PM can induce cardiovascular toxicity [7]. A recent global disease-burden study also indicated that fine PM was associated with risks of low birth weight, preterm birth, and neonatal and infant mortality, particularly in low- and middle-income countries [8].

Emerging evidence indicates a link between PM exposure and hypertensive disorders of pregnancy (HDPs), which are among the leading risk factors for maternal and fetal morbidity and mortality. Gestational hypertension (GH) and preeclampsia (PE) belong to the de novo type of HDP, which accounts for over 85% of HDP diagnoses [9]. HDPs can not only become complicated with proteinuria, edema, seizure, and liver and kidney injury, but also result in a series of adverse pregnancy outcomes, such as intrauterine growth restriction, preterm birth, stillbirth placental abruption, and postpartum hemorrhage [10,11]. In addition, HDPs have grown to become the second leading cause of maternal death in China. Therefore, HDPs are a significant public health problem in China in terms of maternal health [12]. While the etiology of HDPs is still elusive, in recent years an increasing number of researchers have focused on the effects of the environment on cardiovascular health. They have hypothesized that fine PM could result in HDPs via vascular constriction, inflammation, and oxidative stress. However, findings for an association between fine PM and HDPs are inconsistent. A recent Chinese study showed that fine PM exposure during the first trimester was associated with the risk of HDPs [13]. On the other hand, an American study indicated that fine PM exposure increased the development of HDPs during the second trimester rather than the first trimester [14]. This inconsistency may originate from differing study populations and exposure concentrations. Moreover, it has become imperative to draw attention to fine PM exposure before pregnancy. Based on the Developmental Origins of Health and Disease paradigm, the window of sensitivity to fine PM exposure could begin as far back as the preconception period [15]. There is an increasing consensus that preconception exposure to environmental contaminants can affect well-being in pregnancy. Several studies have indicated that air pollution exposure during the preconception period is associated with a risk of termination of pregnancy [16] and gestational diabetes mellitus [17]. The majority of previous studies only focused on the association between air pollution and maternal health in either specific trimesters or the total pregnancy [18,19]. The dynamic association between fine PM exposure and HDPs at different gestational weeks remains to be investigated in Chinese populations. To our knowledge, no study has assessed the association between weekly fine PM exposure, starting during the preconception period, and HDPs, especially de novo HDPs. If an association is shown, preconception precautions for fine PM exposure will become essential practice for lowering the risk of HDPs.

In this retrospective birth cohort study, we collected ambient data on PM exposure and applied a distributed-lag nonlinear model (DLNM) to examine the association between weekly PM exposure during the period from preconception to delivery and de novo HDPs. Additionally, we evaluated sensitivity lag windows for fine PM for HDPs across different sociodemographic strata.

## Methods

### Study Population

The study participants were pregnant women who were part of the Peking University Retrospective Birth Cohort for whom data were available in the hospital information system of Tongzhou for the period between January 2013 and December 2017. All the participants received antenatal examinations and gave birth at the Tongzhou Maternal and Child Health Care Hospital, which is the maternal health center for Tongzhou district, Beijing. The sociodemographic and health details of the participants were recorded at the antenatal examinations, including maternal age (<35 years vs ≥35 years), employment status (employed vs unemployed), parity (nulliparous vs multiparous), prepregnancy weight, height, maternal education level (high school or below, junior college, university or above), maternal ethnicity (Han vs non-Han), first day of last menstrual period, and delivery date. Prepregnancy BMI was calculated by dividing weight (in kilograms) by height (in meters squared) and categorized according to the WHO standards for Asian women: underweight (BMI <18.5 kg/m^2^), normal weight (BMI 18.5-23 kg/m^2^), overweight (BMI 23-27.5 kg/m^2^), or obese (BMI >27.5 kg/m^2^) [20]. The study data were extracted from the electronic medical records of Tongzhou Maternal and Child Health Care Hospital and were routinely reviewed by in-house professional data engineers every week.

The inclusion criteria of this study were (1) a single gestation, (2) an available residential address, (3) no history of previous HDPs, and (4) a delivery date before the 42nd gestational week. A total of 43,894 pregnant women met the inclusion criteria. We excluded 21,324 pregnant women who met the following exclusion criteria: (1) preexisting conditions, including metabolic syndrome; hemolysis, elevated liver enzymes, slow platelets; or hypertension before the HDP diagnosis; (2) missing data for delivery date; (3) an age at delivery less than 18 years; and (4) missing values for maternal age, ethnicity, education level, employment status, or prepregnancy BMI. Finally, 22,570 women were included in this study. The flowchart in Multimedia Appendix 1 shows the details of participant selection.

### Outcome Measurements

The de novo HDP group was defined as patients who experienced GH or PE during the current pregnancy. The normotensive group was defined as those free of any diagnosis of GH or PE and without a history of hypertensive disorders. Information on the history of hypertensive disorders was obtained from initial antenatal examination records. GH or PE diagnoses were made by obstetricians in accord with the Chinese Clinical Practice Guidelines, which are consistent with the guidelines of developed countries [21]. International Classification of Diseases–10 (ICD-10) codes were used to define GH and PE (the ICD codes for GH and PE are described in our previous study [22]). Medical records containing information related to GH and PE were extracted for double confirmation of the diagnosis and to obtain the week of disease diagnosis.

### Assessment of Ambient PM Exposure, Temperature, and Relative Humidity

The pregnant women’s residential addresses were collected at the initial antenatal examination. Each residential address was transformed into longitude and latitude values to determine the concentration of PM_2.5_ or PM_1_ in the center of the nearest 1 km × 1 km area on a grid. Daily PM concentrations were calculated at a spatial resolution of 1 km × 1 km in Beijing during the study period using satellite remote sensing, meteorology, and land-use information. Details of the calculations and validation of PM daily concentration measurements have been reported previously [23]. Data for daily temperature and relative humidity during the study period were obtained from the National Oceanic and Atmospheric Administration [24]. Average daily values for PM, temperature, and relative humidity from preconception (12 before gestation) to delivery (42 gestational weeks) were used to assess atmospheric exposure in our analysis.

### Ethical Considerations

The study was approved by the Institutional Review Board of Peking University Health Science Center (IRB00001052-21023). All the study participants provided written consent. The study data were anonymized to protect the privacy of the study participants.

### Statistical Analyses

The Student *t* test (2-tailed) and chi-square test were applied to assess differences in the distribution of characteristics between the subjects with HDPs and those who were normotensive. The relationships among PM_2.5_, PM_1_, temperature and relative humidity were assessed with the Pearson correlation test.

We followed the weekly exposure to PM_1_ and PM_2.5_ from the 12th week before gestation to the 42nd gestational week [25]. Cox proportional hazard models with a DLNM were used to estimate the association between the weekly PM_2.5_ and PM_1_ exposures and the hazard of de novo HDPs. The basis of the Cox proportional hazard model is the proportional hazards assumption, which means that all the study participants had the same hazard function. Our study complied with this assumption, which means that the ratio of the hazards for any 2 individuals was constant over time [26]. A DLNM was adopted to assess the effects of weekly PM exposures on hazards for HDPs by the “cross basic” function (cb) by constructing a 2D cross matrix that included exposure doses and time to control for the lag effect of PM exposure and the nonlinear relationship of exposure–reaction. A natural spline with 3 degrees of freedom was used to further accommodate the nonlinear effects of PM exposure, temperature, and relative humidity. The maximum number of lag weeks was set at 54 (from the 12th week before gestation to the 42nd gestational week). The lag effect of PM exposure was evaluated by the model as follows: ln(h(t,X)/h0(t)) = *β*1*Zt + *β*2*ns(temperature,3) + *β*3*ns(relative humidity,3) + *β*4*covariables. “ln(h(t,X)/h0(t))” indicates the hazard of de novo HDPs at the specific lag week (t), and the filed event is de novo HDP, noted as X. Zt represented the cross matrix of PM at specific lag weeks. *β*2 and *β*3 are the regression coefficients for temperature and relative humidity with 3 degrees of freedom in the natural spline function. β4 is the coefficient for the covariables, including maternal age, employment status, prepregnancy BMI, maternal education level, parity, maternal ethnicity, and conceptional year.

We also performed several sensitivity analyses to test the consistency of the main results. First, we further used natural splines with 4 and 5 degrees of freedom in the cross matrix to assess the hazards of de novo HDPs. Second, considering the clinical differences between GH and PE, we repeated our analyses excluding GH patients to explore the independent association between PM and PE. Meanwhile, stratified analyses were conducted across strata for differences in employment status, parity, and maternal education level.

All statistical analyses were performed with R (version 4.0.0; R Foundation for Statistical Computing). A 2-sided *P* value <.05 was considered a significant difference.

## Results

### Characteristics of the Participants

There were 1520 participants with de novo HDPs and 21,050 normotensive participants in this study. Table 1 shows the characteristics of the participants. The mean maternal age of the pregnancies was 29.1 (SD 4.1) years. There were 3802/22,570 (16.8%) unemployed women, and 21,222/22,570 (94%) women of Chinese Han ethnicity. A higher percentage of de novo HDPs was observed in the follow sociodemographic strata: low education level, primiparity, and prepregnancy overweight or obesity.

**Table 1:** Characteristics of mothers in the birth cohorts.

Characteristics			Total (n=22,570)		Normotensive (n=21,050)		Hypertensive disorders of pregnancy (n=1520)		*P* value
Maternal age (years), mean (SD)			29.1 (4.1)		29.1 (4.1)		29.4 (4.4)		.13
**Maternal age groups (years), n (%)**									<.001
	Younger than 35	20,126 (89.2)		18,812 (89.4)		1314 (86.4)			
	35 or older	2444 (10.8)		2238 (10.6)		206 (13.6)			
**Maternal ethnicity, n (%)**									.80
	Han	21,222 (94)		19,790 (94)		1432 (94.2)			
	Non-Han	1348 (6)		1260 (6)		88 (5.8)			
**Parity, n (%)**									<.001
	Primiparous	15,320 (67.9)		14,222 (67.6)		1098 (72.2)			
	Multiparous	7250 (32.1)		6828 (32.4)		422 (27.8)			
**Maternal education level, n (%)**									<.001
	Low	6890 (30.5)		6357 (30.2)		533 (35.1)			
	High	15,680 (69.5)		14,693 (68.8)		987 (64.9)			
**Employment status, n (%)**									.80
	Employed	18,768 (83.2)		17,501 (83.1)		1267 (83.4)			
	Unemployed	3802 (16.8)		3549 (16.9)		253 (16.6)			
**Prepregnancy BMI, n (%)**									<.001
	Underweight	2372 (10.5)		2293 (10.9)		79 (5.2)			
	Normal weight	12,032 (53.3)		11,455 (54.4)		577 (38)			
	Overweight	6490 (28.8)		5901 (28)		589 (38.8)			
	Obesity	1676 (7.4)		1401 (6.7)		275 (18.1)			

### Characteristics of PM Exposure

**Figure 1:**
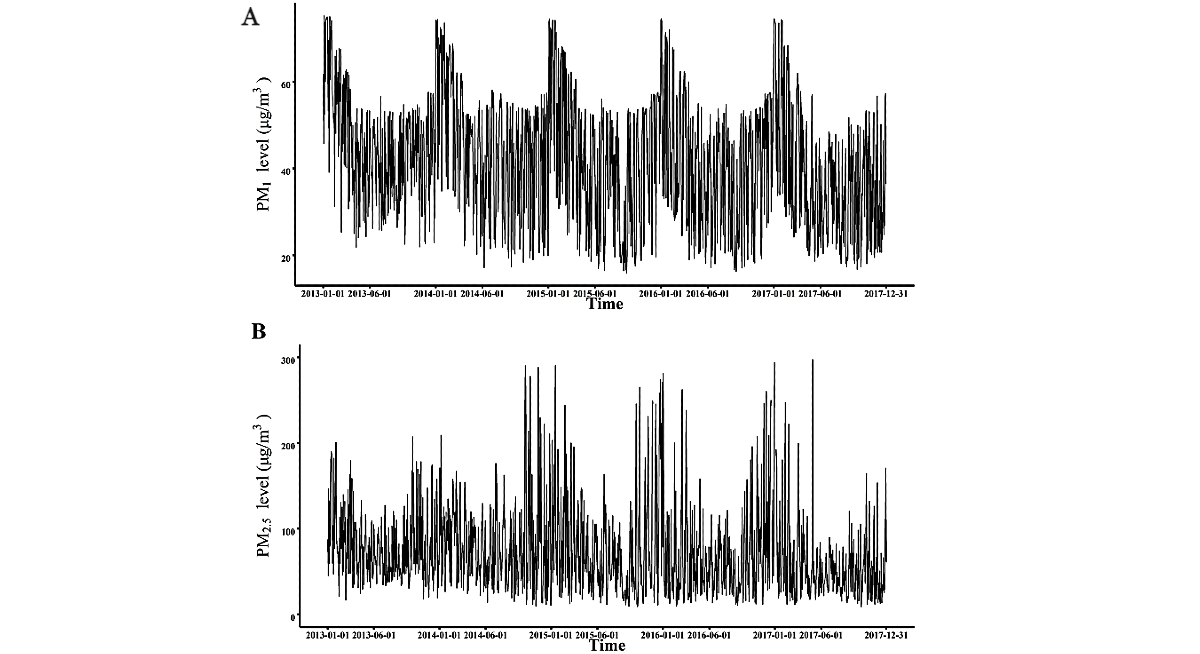
The level of particulate concentration by week. A and B show the daily level of PM_1_ and PM_2.5_, respectively, from 2013 to 2017. PM: particulate matter.

The distribution of PM_2.5_, PM_1_, temperature, and relative humidity is summarized in Multimedia Appendix 2. The average concentration of PM_2.5_ was 74.2 (SD 53.2) μg/m^3^, ranging from 8.5 to 398 μg/m^3^. The concentration of PM_1_ ranged from 16.0 to 75.1 μg/m^3^. Mean temperature and relative humidity were 13.5 °C (SD 11.0 °C) and 54.7% (SD 18.2%). Figure 1 presents the daily fluctuation in PM_2.5_ and PM_1_ concentration from January 2013 to December 2017, revealing seasonal fluctuations in PM_2.5_ and PM_1_ exposure. Meanwhile, PM_2.5_ and PM_1_ were strongly correlated (*r*=0.80). The Pearson correlations among PM_2.5_, PM_1_, temperature, and relative humidity are depicted in Multimedia Appendix 3.

### Association of Weekly Exposure to PM With HDPs

**Figure 2:**
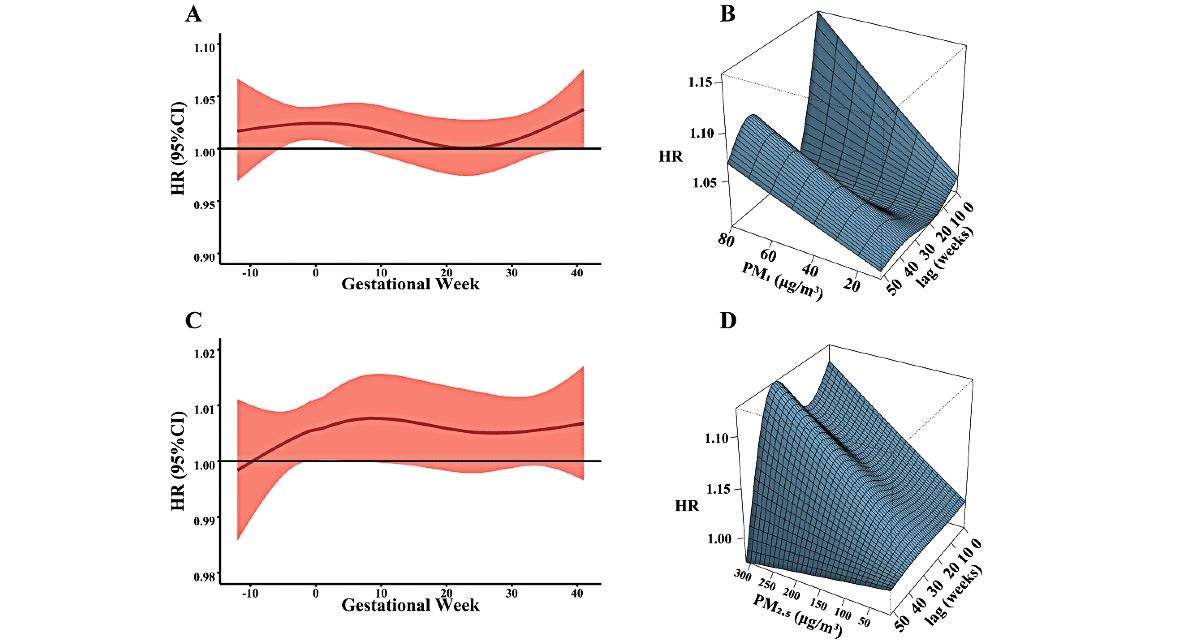
The lag effect of PM on hypertensive disorders of pregnancy in 2D and 3D plots. A and B show the association between PM_1_ and de novo hypertensive disorders of pregnancy in 2D and 3D plots, respectively. C and D show the association between PM_2.5_ and de novo hypertensive disorders of pregnancy in 2D and 3D plots, respectively. HR: hazard ratio; PM: particulate matter.

Figure 2 shows that a 10 μg/m^3^ increase in PM_1_ and PM_2.5_ was associated with de novo HDPs in 2D and 3D plots. The hazard of de novo HDPs was associated with PM_1_ exposure from the 5th week before gestation to the 6th gestational week, and the strongest effect of PM_1_ exposure was observed in the 2nd gestational week (HR 1.024, 95% CI 1.007-1.042; Figure 2A). Figure 2B depicts the effects of PM_1_ exposure on de novo HDPs with different exposure levels and different lag weeks, with a significant single peak by lag week from preconception to the first trimester (from lag 36 weeks to lag 47 weeks). For PM_2.5_, the significant lag window was from the 1st week before gestation to the 6th gestational week and the maximum lag effect of PM_2.5_ was found in the 6th gestational week (HR 1.007, 95% CI 1.000-1.015, Figure 2C). Similarly, we also observed a significant single peak from lag 36 weeks to lag 43 weeks when we assessed the association between PM_2.5_ exposure and de novo HDPs for different exposure doses and lag weeks (Figure 2D).

### Sensitivity Analyses

We conducted two sensitivity analyses to assess the consistency of the sensitivity windows for PM_1_ and PM_2.5_ for de novo HDP hazard. First, using natural splines with 4 and 5 degrees of freedom for the lag constraint, we observed similar results, that is, the significant lag windows for PM_1_ and PM_2.5_ spanned the preconception period and the first trimester (Multimedia Appendix 4). Furthermore, we excluded patients with GH to explore the independent association between PM and PE. Multimedia Appendix 5 shows a result consistent with our primary analyses, that is, that the lag windows for PM_1_ and PM_2.5_ spanned the preconception period and the first trimester.

### Stratified Analyses

**Figure 3:**
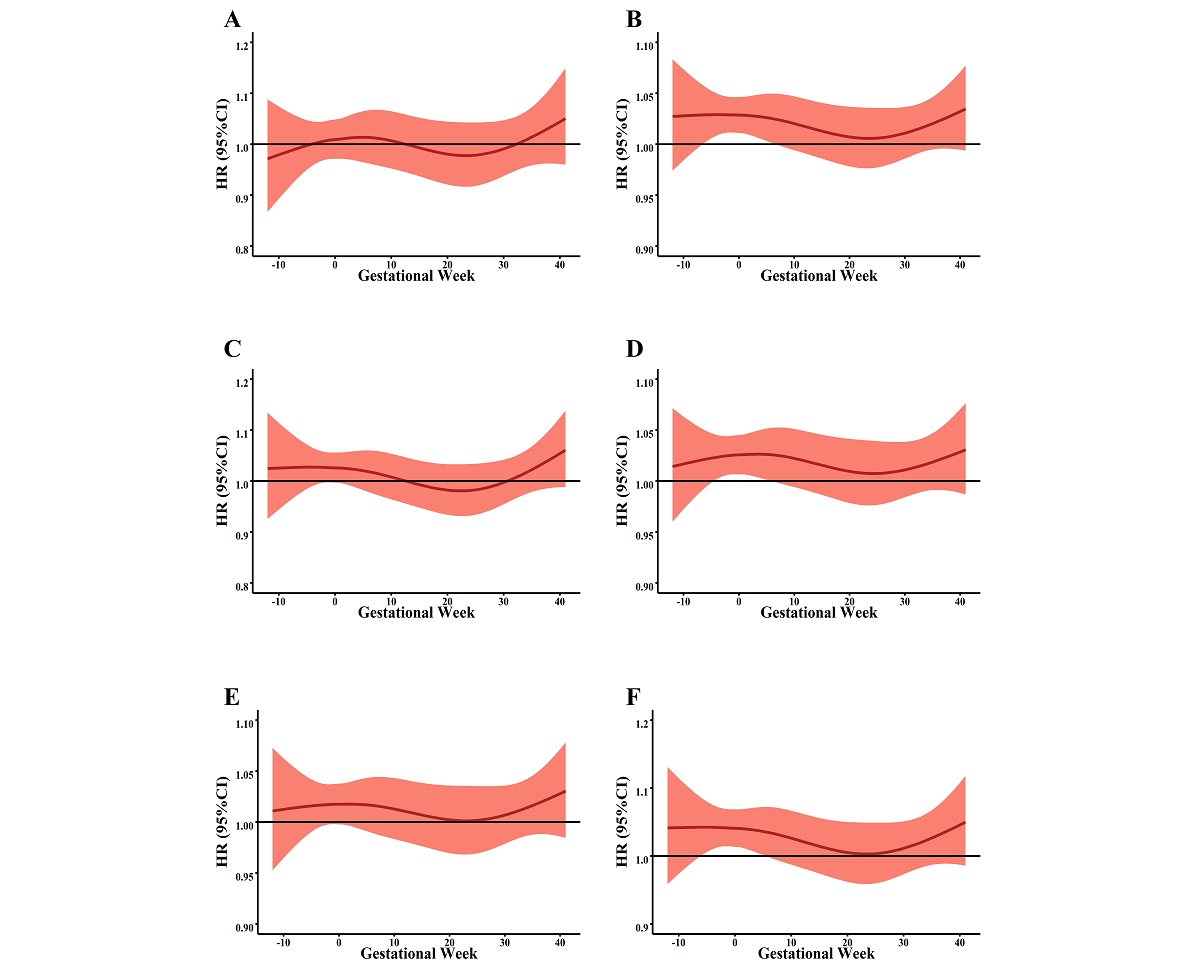
The stratified associations between PM_2.5_ and de novo hypertensive disorders of pregnancy for different maternal characteristics. A and B show the association in unemployed and employed women, respectively. C and D show the association in multiparous and primiparous women, respectively. E and F show the association in women with higher and lower education status, respectively. HR: hazard ratio; PM: particulate matter.

**Figure 4:**
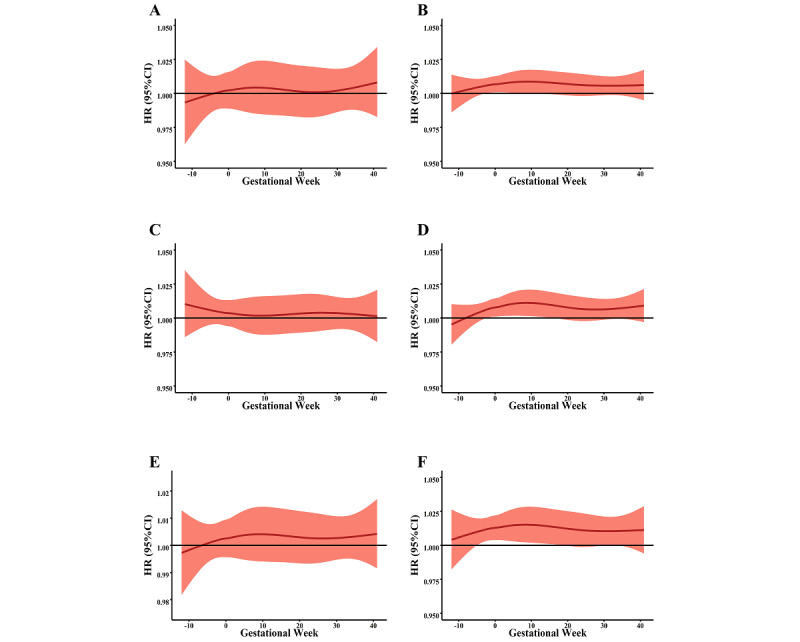
The stratified associations between PM_1_ exposure and de novo hypertensive disorders of pregnancy for different maternal characteristics. A and B show the association in unemployed and employed women, respectively. C and D show the association in multiparous and primiparous women, respectively. E and F show the association in women with higher and lower education status, respectively. HR: hazard ratio; PM: particulate matter.

In the stratified analyses, we estimated the effects of PM on de novo HDPs across differences in employment status, parity, and maternal education level. In terms of PM_1_, a significant sensitivity window for de novo HDPs was observed for employed, primiparous, and low educational status women (Figure 3). Similar findings are shown in Figure 4: women who were employed, primiparous, or had a low education level were more sensitive to PM_2.5_ exposure across the preconception period and the first trimester.

## Discussion

### Principal Results

This study found that the significant lag windows for PM_1_ and PM_2.5_ were from the 5th week before gestation to the 6th gestational week and the 1st week before gestation to the 6th gestational week, respectively, in terms of the de novo HDP hazard. The findings remained robust in sensitivity analyses, which reexamined the associations using different degrees of freedom and after excluding patients with GH. Stratified analyses indicated that pregnant women who were employed, primiparous, or had a lower education level were more sensitive to PM_1_ and PM_2.5_ exposure in the prepregnancy period and the first trimester.

In recent years, numerous studies have evaluated the association between air pollution and cardiovascular health during pregnancy. However, there has been no study to assess the adverse effect of preconception air pollution on gestational health in terms of de novo HDPs. We adopted a time-serial study design to explore the weekly effects of PM_1_ and PM_2.5_ from the preconception period to delivery. To our knowledge, this is the first study to focus on the detrimental association between PM during both the preconception and gestational periods and de novo HDPs. During the study period, the average exposure to PM_2.5_ was 74.2 μg/m^3^, which is more than 2-fold higher than the recommended maximum level of 35 μg/m^3^ set by the National Ambient Air Quality Standards organization [27]. Moreover, exposure to PM showed seasonal fluctuations, peaking in winter and spring. Considering that our participants were mainly northern Chinese living in Beijing, the capital of China, the high concentration of PM can be attributed to central heating and a dense population.

In this study, the prevalence of de novo HDPs was 6.7% (1520/22,570). However, a lower prevalence (5%) of HDPs was reported in a recent study conducted in Shanghai [13]. In comparison with the Shanghai study, the pregnant subjects in our study were exposed to a higher concentration of PM pollution. Since both Beijing and Shanghai have populations of over 24 million and similar demographic compositions, the prevalence of HDPs is unlikely to have been influenced by differences in industrialization or urbanization between these two megacities. The difference in HDP prevalence might be partially explained by the higher PM exposure in the north due to the climate difference and use of central heating.

In our time-serial study, we observed that the women were sensitive to PM_1_ and PM_2.5_ during the preconception period and the first trimester. Our PM exposure measurements were at the week scale, which is more precise than previous studies that have used the trimester scale. Previous studies have made inconsistent findings on the association between fine PM and HDPs. Among 4 US studies, one in California [28] and one in Florida [14] observed that PM_2.5_ exposure was associated with HDPs during pregnancy. However, studies conducted in Rhode Island [29] and New York [30] failed to observe significant associations. There are some potential explanations for these inconsistent results. First, the latitudinal difference may have influenced the temperature and humidity in the different regions. A recent national cohort study indicated that temperature was positively associated with the risk of HDPs during pregnancy [31]. Second, exposure measurement bias may have resulted in a false-negative association. Third, ethnicity differences can affect the association between PM and HDPs. A national cross-sectional study from 2007 to 2018 showed that there was a racial difference in the prevalence of HDPs in the United States [32]. Our study participants mainly lived in Beijing, and 94% were members of the Chinese Han population. Meanwhile, we described our method for high-precision air pollution measurement in a previous study [23]. Our results are consistent with those of the Shanghai study, which had a similar ethnicity and urbanization profile. Thus, climate, measurement, and ethnicity differences were well controlled in our study.

Furthermore, we used a DLNM model, which allowed an examination of the lag effect of PM on HDPs at the week scale, rather than the previously used trimester scale, to improve the precision of the search for the most sensitive gestational week. We also considered potential bias in the model parameters by testing the consistency of our results with different degrees of freedom. Based on the robust results from our sensitivity analyses, we consider that bias from model parameters was not likely. Additionally, our second sensitivity analysis excluding the patients with GE further showed that exposure to fine PM during the preconception period and first trimester was independently associated with the risk of PE. PE is one of the leading causes of adverse pregnancy outcomes, accounting for 10% to 15% of maternal deaths and 15% to 20% of preterm births [33,34]. Early identification of PE risk in clinical practice remains a difficult problem [35]. Our environmental evidence for an association between fine PM exposure and PE might indicate the need for preconception PE prevention to consider environmental hazards.

Considering the strong link with maternal characteristics and gestational blood pressure [36], we further investigated the effects of PM_1_ and PM_2.5_ on HDPs across different maternal characteristics, including employment status, parity, and education level. Among employed women, exposure to PM was significantly associated with the risk of HDPs. PM exposure also elevated the risk of HDPs in women who were primiparous and had a low education level, which indicates that women with these maternal characteristics have a higher risk of HDPs with exposure to PM.

### Strengths and Limitations

There are several strengths of this study. First, to our knowledge, this was the first to adopt the Cox proportion model with a DLNM to estimate the week-scale sensitivity window to PM for HDPs in a Chinese population. HDPs are a major cause of adverse maternal and prenatal outcomes [37]. However, the identification of risk factors for HDPs has been limited due to complex confounders. Previous studies primarily explored the association between PM and HDPs in a specific trimester [38,39]. Our study analyzed the specific gestational weeks and improved the precision of the sensitivity window. Second, this was the first study to cover the effect of air pollution, starting from the preconception period, on de novo HDPs. Our findings reconfirmed the necessity of focusing on air pollution before pregnancy in terms of de novo HDPs. Third, the large sample size of this study made it possible to determine the effects of PM on subtypes of HDP in stratified analyses.

Some limitations must be noted. First, all the participants were residents of Beijing. The lag windows for PM_1_ and PM_2.5_ should be tested in different regions to increase generalizability. Second, PM is a mixture of types of air pollution. The effects of specific PM components should be further explored in future studies.

### Conclusions

Exposure to PM_1_ and PM_2.5_ was associated with the risk of de novo HDPs. The significant lag windows for PM exposure were between the preconception period and the first trimester. Women who were employed, had a lower education level, or were primiparous were more vulnerable to the effects of PM exposure on the risk of HDPs; more attention should be paid to these groups for the early prevention of de novo HDPs.

## Appendix

Multimedia Appendix 1 Study flow chart.

Multimedia Appendix 2 Distribution of PM_2.5_, PM_1_, temperature and relative humidity.

Multimedia Appendix 3 The correlation between ambient exposure.

Multimedia Appendix 4 The association between PM and de novo HDP with 4 and 5 degrees of freedom for the lag constraint. HDP: hypertensive disorders of pregnancy; PM: particulate matter.

Multimedia Appendix 5 The association between PM_1_ and PM_2.5_, and preeclampsia.

## Abbreviations

DLNM: distributed lag nonlinear model
GE: gestational hypertension
HDP: hypertensive disorder of pregnancy
HR: hazard ratio
ICD-10: International Classification of Diseases–10
PE: preeclampsia
PM: particulate matter
WHO: World Health Organization
