# Supplementary figures and images for: Risk of De Novo Hypertensive Disorders of Pregnancy After Exposure to PM1 and PM2.5 During the Period From Preconception to Delivery: Birth Cohort Study
Source: JMIR Public Health Surveill. 2023 Jan 23;9:e41442. doi: 10.2196/41442 (PMC9903185; doi:10.2196/41442)

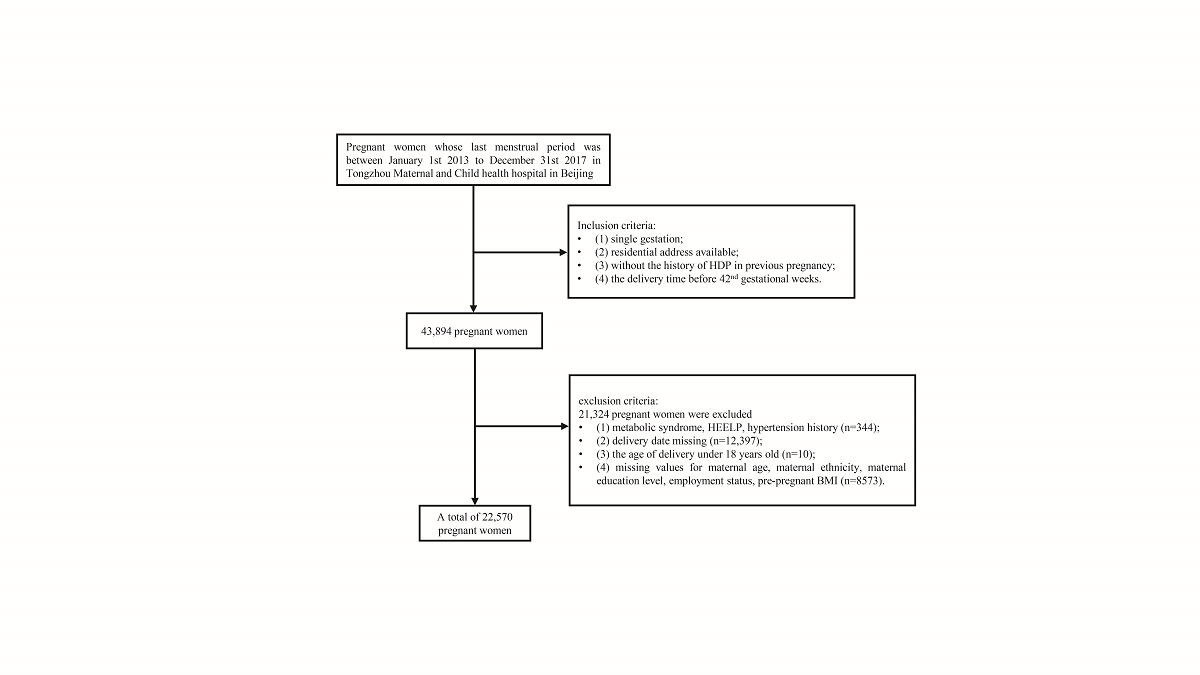

Supplement: Multimedia Appendix 1 [file publichealth_v9i1e41442_app1.png]

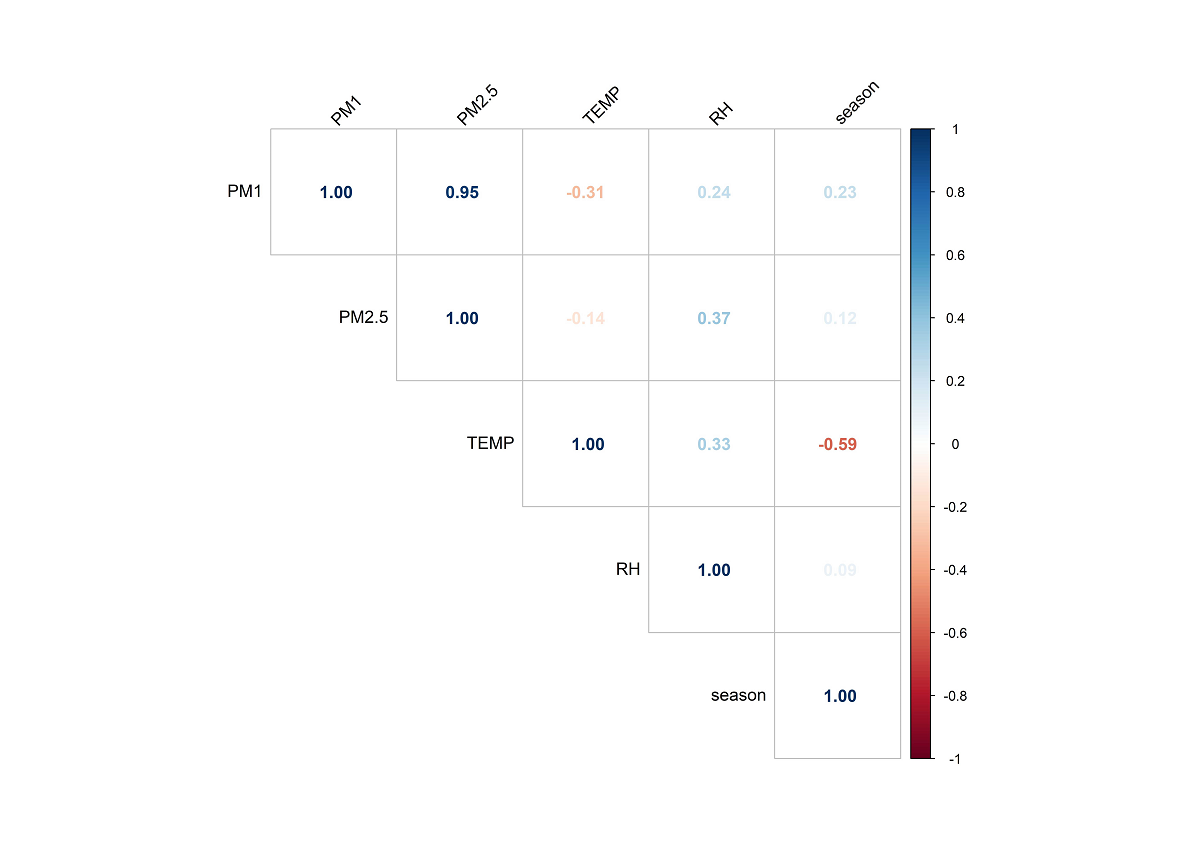

Supplement: Multimedia Appendix 3 [file publichealth_v9i1e41442_app3.png]

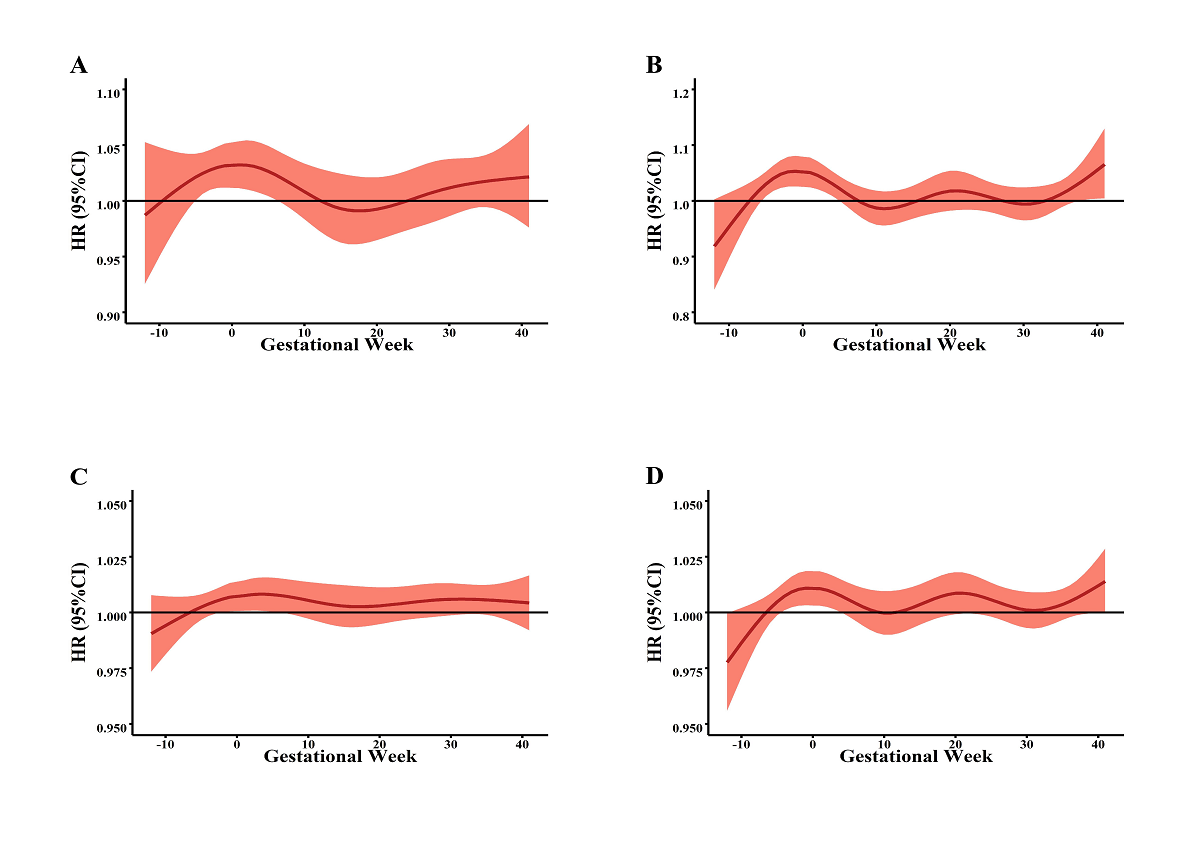

Supplement: Multimedia Appendix 4 [file publichealth_v9i1e41442_app4.png]

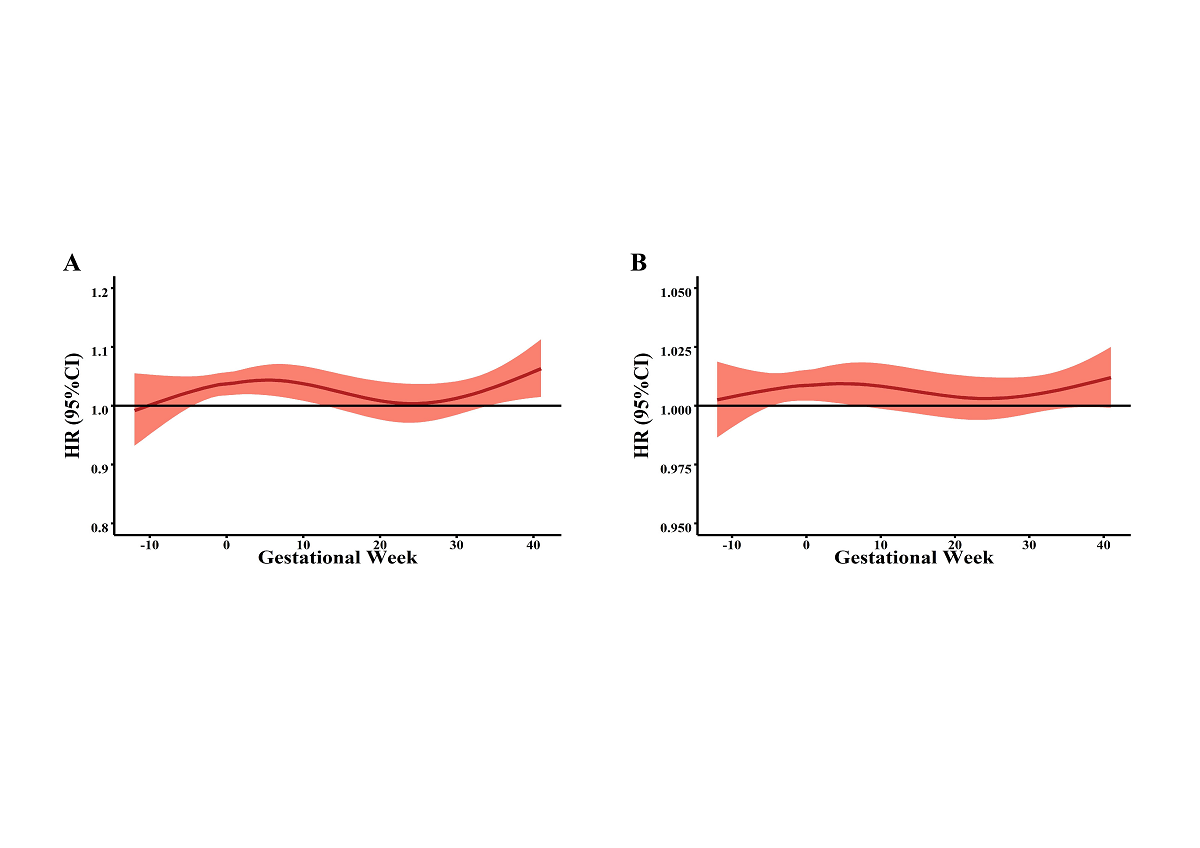

Supplement: Multimedia Appendix 5 [file publichealth_v9i1e41442_app5.png]
