# Supplementary material for: Risk of De Novo Hypertensive Disorders of Pregnancy After Exposure to PM1 and PM2.5 During the Period From Preconception to Delivery: Birth Cohort Study
Source: JMIR Public Health Surveill. 2023 Jan 23;9:e41442. doi: 10.2196/41442 (PMC9903185; doi:10.2196/41442)
Supplement: Multimedia Appendix 2 [file publichealth_v9i1e41442_app2.docx]

| **Multimedia Appendix 2. Distribution of PM_2.5_, PM_1_, temperature and relative humidity** | | | | | | | |
| --- | --- | --- | --- | --- | --- | --- | --- |
| Ambient data | Mean (SD) | IQR | Distribution | | | | |
|  |  |  | Minimum | 25th | 50th | 75th | Maximum |
|  |  |  |  |  |  |  |  |
| PM_2.5_ (μg /m^3^) | 74.2 (53.2) | 59.8 | 8.5 | 37.1 | 60.7 | 96.9 | 398.0 |
| PM_1_ (μg/m^3^) | 41.8 (13.8) | 21.3 | 16.0 | 30.7 | 41.9 | 52.0 | 75.1 |
| Temperature (℃） | 13.5 (11.0) | 20.7 | -14.3 | 3.0 | 14.7 | 23.7 | 32.2 |
| Relative humidity (%) | 54.7 (18.2) | 29.6 | 12.9 | 39.3 | 54.9 | 68.9 | 92.7 |
| PM: particulate matters, IQR: inter-quartile range | | | | | | | |
